# Evidence for positive selection on Mycobacterium tuberculosis within patients

**DOI:** 10.1186/1471-2148-4-31

**Published:** 2004-09-09

**Authors:** Mark M Tanaka

**Affiliations:** 1 School of Biotechnology and Biomolecular Sciences, University of New South Wales, NSW 2052, Australia

## Abstract

**Background:**

While the pathogenesis and epidemiology of tuberculosis are well studied, relatively little is known about the evolution of the infectious agent *Mycobacterium tuberculosis*, especially at the within-host level. The insertion sequence IS*6110 *is a genetic marker that is widely used to track the transmission of tuberculosis between individuals. This and other markers may also facilitate our understanding of the disease within patients.

**Results:**

This article presents three lines of evidence supporting the action of positive selection on *M. tuberculosis *within patients. The arguments are based on a comparison between empirical findings from molecular epidemiology, and population genetic models of evolution. Under the hypothesis of neutrality of genotypes, 1) the mutation rate of the marker IS*6110 *is unusually high, 2) the time it takes for substitutions to occur within patients is too short, and 3) the amount of polymorphism within patients is too low.

**Conclusions:**

Empirical observations are explained by the action of positive selection during infection, or alternatively by very low effective population sizes. I discuss the possible roles of antibiotic treatment, the host immune system and extrapulmonary dissemination in creating opportunities for positive selection.

## Background

How actively do populations of *Mycobacterium tuberculosis *cells undergo adaptive evolution on the spatial and temporal scales of individual infections? On the one hand, the long generation time and limited sequence diversity of this organism might suggest a slow pace of adaptive evolution. On the other hand, the rapidity and ease with which antibiotic resistance is generated during infection suggests otherwise. The physiology and immunology of tuberculosis pathogenesis have been well studied. The infectious agent *M. tuberculosis *is known to invade and replicate within alveolar macrophages. There is a spectrum of responses by the immune system, corresponding to the relative involvement of *Thl *and *Th2 *immune cells, which respectively stimulate the cytotoxic response (more effective against infected cells), and the humoral/antibody response (more effective against extracellular pathogens) [1,2]. Some progress has been made in describing the population dynamics of mycobacterial infection quantitatively [3-5]. At the wider spatial and temporal scales of populations, the molecular epidemiology and the evolution of *M. tuberculosis *have been carefully studied. Genotypic data are rapidly accumulating in the molecular epidemiology of infectious diseases. These are usually compiled and summarised to make inferences about the state of an epidemic in a given geographic location, or at the global level. For example, epidemiologists seek to identify risk factors for infection, and to locate particular strains that are especially transmissible or pathogenic [6-8]. The evolutionary history of *M. tuberculosis *has also been characterised. For example, it has been argued that the limited variation at the nucleotide is due to a recent population bottleneck [9], and that the common ancestor of *Mycobacterium bovis *and *M. tuberculosis *may well have been a human rather than bovine pathogen [10].

Less understood is the evolution of *M. tuberculosis *at the cellular level *inside *bodies. There has been little integration of the genetic information from markers with the population genetics of the bacterial population within hosts. In this article, I examine data collected for the purposes of molecular epidemiology to present three lines of evidence supporting the action of positive selection on *M. tuberculosis*. The data come from the marker IS*6110*, which is currently the standard method of typing tuberculosis isolates. These genotypic data will be considered under assumptions of neutrality, and then under the assumption that positive selection is acting. The case for the action of selection is based on the following three arguments.

• Under the assumption of neutrality, the observed mutation (or transposition) rate of the genetic marker IS*6110 *is unusually high; the estimated mutation rate is lower if selection is acting.

• The observed times associated with change are too low to be explained by neutrality; positive selection lowers the expected substitution time.

• The observed level of polymorphism is too low to be explained by neutrality.

## Results: Models and observations

In each of the following sections a comparison is made between the strictly neutral model and a generalised model including selection through a single parameter *s *(described in the Appendix). Although the analyses start with strict neutrality (*s *= 0) in each argument, alleles for which *s *< 1/*N*, where *N *is the effective population size, can be considered *nearly neutral*, in that the effects of drift outweigh the force of selection [11]. In each case, explaining observations in this range of selective coefficients requires very low effective population sizes.

### Transposition rates of IS6110

When genetic mutations are selectively neutral, the substitution rate is equal to the mutation rate [11]. In the present case, the *within-host *substitution process is of interest. Rosenberg *et al. *[12] determined the within-host substitution rate of the *IS6110 *marker to be around 0.00184 to 0.0390 events per copy per year, with the maximum likelihood estimate at 0.0287. Under neutrality, therefore, this rate corresponds to a *per insertion *mutation rate of *μ*_*i *_~ 7.9 × 10^-5 ^events per site per generation, assuming a generation time of 1 day in active infections. This figure comes from a measured doubling time of close to 24 hours, based on clinical isolates grown in human monocyte cultures and in culture media [13-15]. Rates of point mutation (events *per nucleotide *per generation) are usually in the vicinity of 10^-9^. In mutator strains, that is, genomes in which the DNA repair machinery is damaged, leading to elevated mutation rates, the mutation rate rises orders of magnitude, up to ~10^-7 ^– 10^-6 ^[16]. The mutation rate of IS*6110 *under neutrality therefore seems suspiciously high, although this is only "circumstantial evidence", since it is not inherently problematic. Indeed, mutation rates as high as 10^-4 ^per element per generation have been measured for IS*10 in vitro *[17]. Nevertheless, if positive selection is allowed the estimated mutation rate decreases. Leaving aside the complicating influence of clonal interference [18], the rate of substitution is

**Figure 1:**
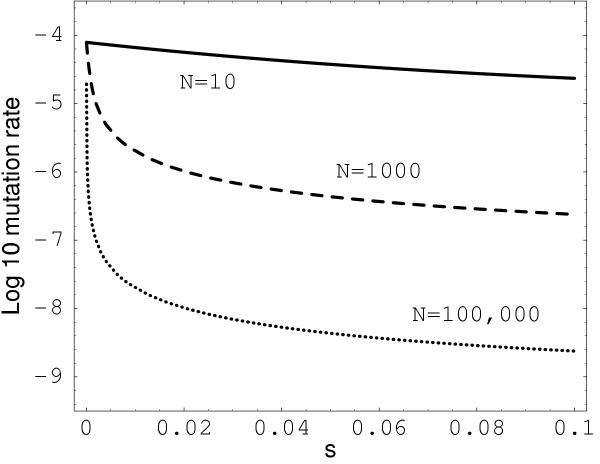
**Estimate of mutation rate when positive selection is acting. **The estimate  is plotted on a logarithmic scale in base 10. Solid curve: *N *= 10; Dashed; *N *= 1000; Dotted: *N *= 10^5^.

*K *= *uN μ*_*i *_    (1)

where *u *is the probability of fixation of a mutant, *μ*_*i *_is the mutation rate and *N *is the population size [11]. An estimate of the mutation rate when mutants have advantage *s *is  = *K*/(*uN*). The diffusion model of drift provides an expression for *u *as a function of the population size *N *and selective coefficient *s *(see the Appendix). Figure 1 plots  over *s *for a few different values of *N*. In each curve the estimated mutation rate decreases as the selective coefficient rises. According to this analysis, lower mutation rates are possible when there is some selection and a large population size, or when selection is strong and the population size is small. Note that the estimated mutation rate remains high if mutations are nearly neutral.

### Fixation times

Various studies have measured the stability of IS*6110 *as a genetic marker by examining genotypes of serial isolates from patients with persistent infection. A small number of changes in the genotypes between serial isolates indicates a stable marker. Differences in genotypes due to exogeneous reinfection by unrelated strains are excluded from consideration. In the data of Niemann *et al. *[19] and Rosenberg *et al. *[12], the median time interval associated with changes in IS*6110 *genotypes from serial samples of *M. tuberculosis *is 212 days, and the maximum is 683 days. Because the second sample is taken some time after fixation of the mutant, the actual substitution times are unknown, but they were clearly all under 683 days. I will now show that the expected substitution times under strict neutrality are well in excess of this value. 

Let us start with the assumption that the expected time for substitution to occur is the average time taken for the successful mutant to appear plus the time taken for that mutant to reach fixation conditional on its eventual fixation. (I will later drop the assumption about waiting for the mutant to appear). The average appearance time is 1/(*μNu*) = 1/*μ *since *u *= 1/*N *under strict neutrality. The average time for a successful neutral mutant to reach fixation is 4*N *generations. The mutation rate of interest in this context is the rate *per genome *per generation, since what is of concern is whether any of the elements in a given genome produce change. For simplicity, assume that the genomic mutation rate scales linearly with copy number. (At the resolution of this analysis, this is a reasonable approximation.) Considering a typical strain has 10 copies of the IS element, the relevant mutation rate here is *μ *= *μ*_*i *_× 10 = 7.9 × 10^-4^. Therefore, for *N *= 10, 10^3^, 10^5^, the expected substitution times are roughly 1300, 5300, 4 × 10^5 ^generations, respectively. With the generation time set to one day, the upper bound of observed substitution times was 683 generations, which is well below theoretical expectations.

Now consider the possibility of positive selection under two alternative conservative assumptions. The earlier assumption that there are no successful mutants at the time of the first sample is favourable to the parental strain. A more conservative approach (favouring mutants) would be to say that the mutant destined to reach fixation appears exactly at the time of the first sample. We can then ask how long it takes on average for this mutant to reach fixation if it is positively selected. An even more conservative model would be that not only is the successor strain present at the time of the first sample, but is present at a frequency of 30%. Furthermore, let us say the subdominant strain only needs to be at 70% at the time of the second sample to be considered to have replaced the parental strain.

A model of the sojourn times of alleles in populations conditional on fixation must now be specified. Again using the diffusion model of drift (see Appendix), the mean time spent by a mutant in the range of frequencies (*a, b*) (provided *a *is greater than the initial frequency), conditional on fixation, was found by Ewens [20] and by Maruyama [21] to be



**Figure 2:**
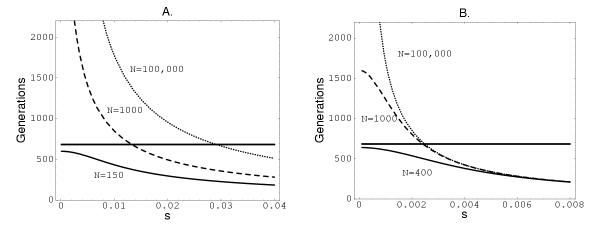
**Mean sojourn times as functions of selective coefficient *s*, for different values of *N*. **Left: from *a *= 1/*N *to *b *= 1 - 1/*N*; Right: from *a *= 0.3 to *b *= 0.7.

Figure 2 shows the two conservative models, corresponding to two different boundary values for (*a, b*). Even in the extremely conservative model shown in the right-hand plot, the effective population size must be below 400 in order to explain the observed substitution times under strict neutrality. The data are difficult to account for even in terms of nearly neutral mutations (*s *< 1/*N*) and an effective population size of *N *= 1000. The alternative explanation is that the effective population size is larger, but positive selection is acting to make changes sweep through the population faster.

### Polymorphism

Many analyses of pathogen genotypes assume isolated strains to be clonal, that is, to be monomorphic. This assumption has been scrutinised by De Boer *et al. *[22], who showed that, in fact, a large proportion (93%) of *M. tuberculosis *isolates are monomorphic using IS*6110 *as the marker. They also show that the limits of detection of a second strain are around frequencies of 0.1 to 0.3. More sensitive instruments and refined genotyping procedures are likely to reveal greater polymorphism. The current information can be used, however, to study the population of the organism in hosts by using ranges of *detectable polymorphism*. In this section, two ranges will be considered in examining predictions from models: first, 0.1 to 0.9, and second, 0.3 to 0.7.

The polymorphism argument rests on the assumption that the isolates reported in [22] can be viewed as a random sample from a set of populations in mutation-drift equilibrium. It should be noted that because the isolate represents a sample of cells from the patient, it presumably does not always reflect the diversity of cells in the greater within-host population. Thus the polymorphism or heterogeneity observed from isolates is an underestimate of the actual levels.

Wright [23] found the stationary probability distribution of allele frequencies under the diffusion model with mutation and two alleles. Let *f*(*x*) be the probability density function of this distribution and *F(x) *be the cumulative probability function *F(x) *(see Appendix). The probability that a given population (patient) is between frequencies *a *and *b *(where *a *<*b*) is



This quantity can be alternatively interpreted as the proportion of populations observed to be polymorphic according to the detection limits set by (*a, b*).

**Figure 3:**
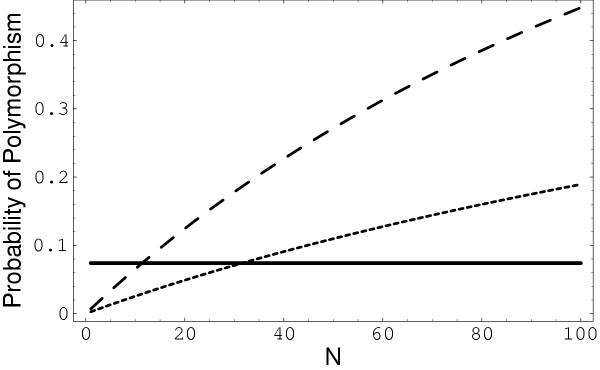
**Probability of detecting polymorphism in the absence of selection, as a function of *N*. **Two different ranges of detectable polymorphism were used. Dashed curve: (0.1, 0.9); dotted: (0.3, 0.7). We use *μ *= 7.9 × 10^-4^. The horizontal bar indicates the observed fraction of polymorphic populations (0.074) from de Boer *et al*. [22].

First consider the neutral case. When there is no selection (*s *= 0), the distribution described by *f(x) *is a Beta distribution. Figure 3 shows the probability of an isolate being scored as a polymorphic population, using two alternative detectable polymorphism ranges (*a, b*) = (0.1, 0.9) and (0.3, 0.7), and a mutation rate of *μ *= 7.9 × 10^-4 ^per cell per generation.

**Figure 4:**
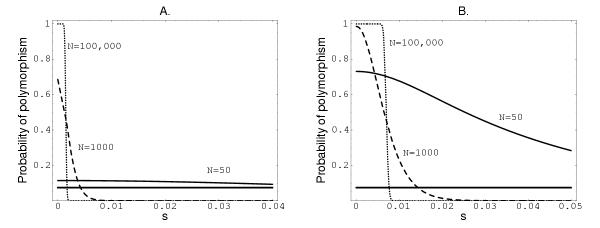
**Probability of polymorphism as a function of *s*. **Left: the detection threshold is set at 0.3; Right: the detection threshold is set at 0.1. The mutation rate is set to *μ *= 7.9 × 10^-4^

Next, consider the model that includes selection. For the two detectable polymorphism ranges, Figure 4 shows how selective coefficient *s *and effective population size *N *are related to the probability of observing polymorphism. As *s *increases, the predicted polymorphism decreases dramatically, particularly for large *N*. Again, an explanation of the observed level of polymorphism is only possibly by setting *N *to be extremely low.

## Discussion

The three lines of evidence presented in this article suggest positive selection on *M. tuberculosis *within hosts. There are, however, limitations to these analyses. In the first argument there is no inherent problem with finding transposition rates that are high. In the second argument, it is possible to lower the effective population size far enough to explain the speed of substitution. In the third argument, 1) the bacterial populations sampled in [22] might not be close to mutation-drift equilibrium, 2) the sampled cells might not reflect the true diversity of the bacterial population in a patient, and 3) the levels of polymorphism again may be explained by very low effective population sizes. Consistency with observations nevertheless requires *N *values of around 100 or lower, which seems grossly at odds with the usually large census population sizes of bacteria. In mouse models of TB infection, for instance, bacterial loads reach around 10^5 ^– 10^7 ^colony forming units per lung [2,24]. It has been noted, however, that effective population sizes of bacteria can be much lower than actual sizes [11,25].

I will also comment on why I have not attempted to statistically fit the model to data to estimate *N *and *s*. First, from the plots shown here, it is clear that different combinations of the two parameters can explain the observations. This would make it difficult to locate the best fit. Second, although the model can be used to assess the possibility of neutrality in the current context, it cannot adequately serve as a framework for estimation given the intricacies of host-pathogen interactions. Further, adding more parameters to the model would increase the complexity of the analysis beyond what can be sustained by the resolution of the currently available data.

Taken together, the results suggest positive selection, although the evidence is not conclusive. A possible alternative is that the effective population sizes of *M. tuberculosis *within patients are very low due to population structure, background selection, or other factors. If there is indeed detectable adaptive evolution of tuberculosis within patients, what are the sources of selection? Two important candidates are antibiotic treatment and the host immune system. Studies using serial isolates have found no correlation between IS*6110 *genotype instability and (a) drug resistance/susceptibility of the isolate [26,27], (b) *change *in drug resistance status [19] or (c) drug adherence by the patient [26]. It is still possible, however, that the collection of observed changes involve a variety of different genetic loci, with at least some conferring drug resistance, although such events may not be statistically detectable. Further, mutation in drug resistance loci will not necessarily be revealed by a marker. Genetic analysis of isolates of *M. tuberculosis *from the lung lesions of six patients has shown heterogeneity in resistance-associated alleles, but not with respect to IS*6110 *[28].

Alternatively, fingerprint changes may reflect (evolutionary) escape from the immune system. The analysis here hints at low effective population sizes – perhaps the immune system induces a heavy decline in population sizes of *M. tuberculosis *within patients, i.e., bottlenecks – which is overcome by survivors with new genotypes. If the observed patterns are to be explained by severe bottlenecks, the surviving cells are not necessarily better adapted to residing in the host than the parental cells that were eliminated by the immune response. It is noteworthy that Yeh *et al. *[26] found no relationship between HIV status of the patient and genotype instability. This suggests that genetic changes in *M. tuberculosis *are not primarily driven by the immune system. However, the extraordinary ability of *M. tuberculosis *to manipulate the T cell response [2] suggests the role of adaptation to the immune system in the deeper evolutionary history of the organism.

Interestingly, de Boer *et al. *[27] found an association between IS*6110 *change and extrapulmonary disease or pulmonary+extrapulmonary disease and extrapulmonary origin of isolates. Dissemination is a major factor in the pathogenesis of tuberculosis. Since the lungs are the preferred environment of the organism, the new environments outside lungs may create opportunities for adaptive evolution. Adaptive evolution leading to specialisation to tissue types is to be expected. A recent article [29], for example, has found the occurrence of tissue-specific adaptations in *Streptococcus pyogenes *by examining ratios of non-synonymous to synonymous substitution rates (*d*_*n*_/*d*_*s*_).

Is IS*6110 *directly responsible for adaptive mutations? On one hand, the apparently strict asexuality of *M. tuberculosis *implies that all genes good, bad or neutral are tightly linked to each other. It is likely then that IS-induced changes hitchhike to fixation with other mutations that confer advantage to the genome. On the other hand, it has recently been demonstrated that IS*6110 *carries a promoter that can modify the the expression of neighbouring genes, raising the possibility of a direct role for the element in adaptive evolution [30]. Note that changes caused by IS*6110 *can be not only beneficial, but also neutral or deleterious [31].

At the within-patient level, the best studied pathogen is perhaps HIV. While *M. tuberculosis *shares with viruses the characteristic of replicating within cells, a major difference is that mutation rates in viruses are much higher, particularly in retroviruses, which depend on reverse transcriptase (a low-fidelity enzyme) to copy their genomes. Hence, the extent of nucleotide variation of *M. tuberculosis *is not expected to be the same as is commonly observed for example in HIV [32]. There is ongoing controversy among HIV researchers about the role of stochasticity due to low effective population sizes in the evolution of the virus [33-35]. In any case, investigating the ratio of non-synonymous to synonymous substitutions (*d*_*n*_/*d*_*s*_) has established the action of positive selection on HIV within patients [32,36].

In *M. tuberculosis*, the level of polymorphism at synonymous sites has been noted to be extremely low [9]. It would be of interest to measure the ratio of non-synonymous to synonymous polymorphisms in key genes, such as loci conferring resistance to drugs, or those implicated in interactions with the immune system. These *d*_*n*_/*d*_*s *_ratios may provide further insight into the nature of positive selection in *M. tuberculosis*.

## Appendix: Bacteria and the Wright-Fisher process

The analyses here rely on the commonly used diffusion model of drift and selection in a population, based on the Wright-Fisher process [37,38]. It is also possible to use the Moran model, in which at each time step an individual is chosen randomly to reproduce, and then another individual is chosen to die. The individual to die may be the same as the individual that reproduced, but not the offspring. Selection can be incorporated by including differential probability of birth or death for different genotypes. As noted by Ewens [38], the Moran model closely resembles the Wright-Fisher model; the critical difference between the two models arises from differences in the distribution of offspring number. The theory is usually discussed in relation to a diploid population of size *N*_*e *_in which there are 2*N*_*e *_copies of the (autosomal) gene in question. The diffusion model is used here with minor adjustments to describe bacterial populations. Let the number of bacterial cells in a population be *N*. Each mutant appears in the population at frequency 1/*N*. Realistically, population sizes fluctuate and only a subset of cells actively divide. The number *N *should therefore be considered to be the effective rather than the actual population size, which may be much larger than *N*.

### Mean and variance of change

It can be shown that the deterministic dynamics of selection in a haploid model are well approximated by a logistic model. The mean change in frequency *x *of an allele per generation is *m*(*x*) = *sx*(1-*x*), which is identical to the diploid model with additive fitnesses (no dominance) if each copy of the advantageous allele adds *s *to the fitness (see [[37], p. 192]). That is, heterozygotes enjoy a fitness advantage *s *and homozygotes have advantage *2s*.

The variance component *v(x)*, the variance in change of allele frequency per generation, can also be taken from diploid theory. Replacing the diploid model of the random union of gametes with choosing cells randomly from each generation to the next, the effective population size is adjusted according to the distribution of offspring number under a given model of cellular division.

### Binary fission

There are numerous ways to model drift in populations of organisms that reproduce by dividing to produce two daughters [39]. Here, cells are assumed to undergo fission synchronously and daughter cells are chosen randomly at each generation. In the absence of selective effects, the offspring distribution is *p*_0 _= 1/4, *p*_1 _= 1/2, *p*_2 _= 1/4, where *p*_*i *_is the probability of producing *i *offspring. The variance in offspring number here is 1/2 and the variance-effective population size equals *N*/ = 2*N*. Thus in this case, the diploid theory can be directly used as far as *v(x) *is concerned (replacing 2*N*_*e *_with 2*N*). Johnson and Gerrish [39] consider alternative models. These alternatives are associated with different rates at which drift proceeds in a population, and would not affect the qualitative conclusions drawn here.

### Fixation probability

Diffusion models of genetic drift have shown [11] that the probability of fixation of an allele at frequency *p *in a randomly mating diploid population of size *N*_*e *_(with 2*N*_*e *_copies of the gene in question) is



Therefore, using *p *= 1/*N *rather than the usual *p *= l/(2*N*_*e*_) the probability of fixation of a mutant bacterial cell with selective advantage *s *is



Note that when 4*N*s >> 1, *u *~ 4*s*. This agrees with a result of Gerrish and Lenski [18], using a branching process model (rather than the diffusion model) to find the fixation probability under this same model of binary fission. See [39] for discussion of *u *for alternative models.

### Steady state distribution

Let the mutation rates from any genotype to any other be equal (*μ*). As stated above, selection is additive. As shown by Wright [23], the steady state distribution of allele frequency is then given by the density function



See also [37,38] for further details.
